# Warming Treatment Methodology Affected the Response of Plant Ecophysiological Traits to Temperature Increases: A Quantitive Meta-Analysis

**DOI:** 10.3389/fpls.2019.00957

**Published:** 2019-09-06

**Authors:** Dan Wang, Hao Wang, Pengpeng Wang, Tianqi Ling, Wenhui Tao, Zaiqiang Yang

**Affiliations:** ^1^Jiangsu Key Laboratory of Agricultural Meteorology, International Center for Ecology, Meteorology and Environment, Institute of Ecology, Nanjing University of Information Science and Technology, Nanjing, China; ^2^Meteorological Bureau of Chengde City, Chengde, China

**Keywords:** photosynthesis, respiration, controlled experiment, climate warming, meta-analysis

## Abstract

Global mean temperature is expected to significantly increase by the end of the twenty-first century and could have dramatic impacts on a plant's growth, physiology, and ecosystem processes. Temperature manipulative experiments have been conducted to understand the responsive pattern of plant ecophysiology to climate warming. However, it remains unknown how different methodology used in these experiments will affect plants ecophysiological responses to warming. We conducted a comprehensive meta-analysis of the warming manipulative studies to synthesize the ecophysiological traits responses to warming treatment of different intensities, durations, and conducted for different species and under different experimental settings. The results indicated that warming enhanced leaf dark respiration (R_d_) and specific leaf area (SLA) but decreased net photosynthetic rate (A_net_) and leaf nitrogen content (LN). The positive and negative effects of warming on R_d_ and A_net_ were greater for C_4_ species than C_3_ species, respectively. The negative effect of warming treatment on A_net_ and LN and the positive effect on R_d_ were more evident under >1 year warming treatment. Negative effects of warming were more evident for plants grown at <10 L pots when experiment duration was longer than 1 year. The magnitude of warming treatment had a significant impact on most of the parameters that were investigated in the study. Overall, the results showed that warming effects on plant ecophysiological traits varied among different response variables and PFTs and affected by the magnitude of temperature change and experimental methodology. The results highlight the need for cautiously selecting the values of plant ecophysiological parameters in forecasting ecosystem function changes in future climate regimes and designing controlled experiments to realistically reflecting ecosystems responses to future global warming.

## Introduction

Based on the current trends in fossil energy production and use, deforestation, and population growth, it is expected that the increase of global mean surface temperatures for 2081–2100 relative to 1986–2005 is projected to be in the ranges of 0.3 to 1.7°C (RCP2.6), 1.1 to 2.6°C (RCP4.5), 1.4 to 3.1°C (RCP6.0), and 2.6 to 4.8°C (RCP8.5), which will have dramatic effects on economics, agriculture, and environment (AR5, IPCC, 2013). Plant traits are sensitive to climate warming and ecologists use plant trait-climate relationships to simulate plant physiology and growth in current and future climate situations (Farquhar and Sharkey, 1982; Wang et al., 2012; Jing et al., 2016). Therefore, understanding the patterns of plant physiological and morphological responses to global warming is of great importance in simulating and predicting the impact of global change on natural systems and agriculture.

Predictions of response to global warming may be derived from experimental and observational studies (Tilman, 1989; Wang et al., 2008, 2018; Knapp et al., 2012). While both types of study are common, relatively few authors have investigated whether they produce similar predictions or reflect reality (Dunne et al., 2004; Knapp et al., 2018). Experimental global change studies are typically limited in scope both spatially and temporally (Rustad et al., 2001). Observational studies often have broader spatial and temporal scales but suffer from a lack of control over covariates in biophysical and biochemical parameters of weather and soil. To minimize the weaknesses of each approach, it has been suggested that more research should explicitly unite observational and experimental work, perhaps by nesting experiments at multiple sites within a larger observational context or through summarized meta-analysis (Dunne et al., 2004; Jing et al., 2016).

Many manipulative experiments controlling physical and environmental factors have been conducted around the world to investigate the potential effects of global change on plants and terrestrial ecosystems (Sage and Kubien, 2007; Rustad, 2008; Wang et al., 2018). However, the methodology used in these experiments was different in their research settings, treatment intensities and durations and targeted species. The impact of short-term vs. long-term warming on plants traits would probably be different due to plants' acclimation capacity in photosynthesis, respiration and other physiological processes and these impacts would vary among different plant functional types (PFTs) under natural or controlled settings (Smith and Dukes, 2017). Plants' physiological and morphological responses to short-term warming treatment, however, are often used to parameterize the sub-models of photosynthesis, stomatal conductance, and respiration in plant growth and terrestrial ecosystem models, which would likely unrealistically simulate plant energy, carbon, and water fluxes in the long term. Indoor or outdoor settings and pot sizes could also affect the magnitude of ecophysiologial responses to temperature increase by implicating root growth and plant above-ground and below-ground tissue interactions (Arp, 1991). To accurately predict the impacts of climatic change and develop proper adaptive agricultural management practices, it is imperative to understand how temperature changes of different intensities and duration and changes manipulated under different experimental settings affect photosynthetic carbon gain, loss and allocation through a comprehensive analysis of relevant studies.

Previous research and meta-analyses have indicated that global warming will promote plant photosynthesis, dark respiration, leaf nitrogen content, specific leaf area, and other metabolisms (Poorter et al., 2012). It has been reported that the modulation of leaf traits and trait relationships by site climatic properties was modest (Wright et al., 2005). However, the modulation of leaf traits by warming treatment of different intensities and duration has not been extensively analyzed. Understanding how these processes vary among different species and plant functional types is a major goal for plant ecology and crucial for modeling how nutrient fluxes and vegetation boundaries will shift under global warming. The effect of the intensities and the treatment duration of global warming manipulative experiments on the plant physiology and growth among different plant functional groups, however, remain unclear. Therefore, the main objective of this study was to investigate the effects of global warming treatment with different magnitudes and durations on plant response in ecophysiological traits. Specifically, we aim to: (1) assess the impact of global warming of different magnitudes and durations on plant ecophysiological traits at leaf level; (2) detect the variations of ecophysiological traits response of different plant functional types to warming treatment of different durations; (3) explore the effect of different experimental settings on the response of a plant's traits to global warming. Accordingly, we propose: (1) due to plant acclimation capacity, short-term vs. long-term warming has different impacts on plant traits, with short-term warming having a more stimulating effect on the physiological functions of plants; (2) different experimental facilities may change the response of plants traits to warming treatment. To test these hypotheses, we conducted a comprehensive meta-analysis of the warming manipulating studies published from 1980 to 2018.

## Materials and Methods

### Data Collection

Journal articles were searched on the Web of Science database with the keyword “leaf traits & warming,” “leaf traits & temperature increase” and etc. The articles were later cross-checked with review articles and book chapters. The articles were imported into EndNote software and formed a database. All articles about warming effects on leaf traits were screened to ensure that all the articles available were included for the analysis. The articles published from 1980 to 2018 and meeting the following two conditions were included in the analysis: (1) the control group in the experiment was treated at ambient temperature situation; (2) physiological and morphological measurements were performed on both ambient and manipulated groups. Articles were rejected if: (1) plant physiological changes under warming treatments led to death of or severe damage to the plant; (2) there were other stressing factors impacting the warming treatments. Finally, 80 papers meeting the requirements were included in the database (Supplementary Material S1). Data was obtained directly from the table or was extracted using the GetData Graph Digitizer software from the selected articles. In these studies, the magnitude of warming treatment ranged between 0.3 and 25°C, with only two studies showing a warming treatment above 20°C above AT (Supplementary Material S1). Response variables collected from these articles included net photosynthetic rate (A_net_), stomatal conductance (G_s_), leaf nitrogen (LN), dark respiration (R_d_), and specific leaf area (SLA). When A_net_, R_d_, and G_s_ of one species with the same unit were all provided in the study (including measurements conducted on the same leaves/individuals and those across individuals), the R_d_/A_net_, and A_net_/G_s_ in the control and warming treatments were calculated. In addition to the above responsive variables under different treatments, plant species, sample size, growth facilities, and duration of warming treatment were also collected. To ensure the independent nature of the data, we excluded duplicate results collected from the same studies. However, our analyses were not completely independent because individual study often provided data with more than one treatment (e.g., different warming treatment intensities) and/or different response variables. To examine the influence of non-independence of data, we first averaged those data from the same published study by PFTs so that only one comparison was used from a published study for each PFT. Nonetheless, we found that most of the response patterns were unchanged; therefore, all data were used in our study.

### Categorization of the Studies

Temperature treatment was divided into two categories: AT (ambient temperature) and ET (elevated temperature). Plant species were classified into different photosynthetic pathways (C_3_, C_4_, or CAM), growth forms (herb or wood) and economic values (crop or non-crop). Experimental facilities were categorized into indoor (growth chambers or greenhouses) and outdoor (open top chambers or fully-open) settings and <10 L and >10 L growing pots. In our dataset, exposure time (i.e., how long plants were exposed to warming) ranged from <10 days to >10 years. To analyze the possible different responses under various warming durations, we banded the temperature treatment into two categories: short-term (<1 year) and long-term (>1 year). Warming treatments that were applied through air warming were included in the analyses. We listed the species, PFTs information and relevant experimental methodology used in this study (Supplementary Material S1).

### Meta-Analysis Methods

To avoid the adverse effects of different units, we used the response ratio r = X_t_/X_c_ to estimate the magnitude of the effect of warming treatment, where X_t_ is the treatment mean and X_c_ is the control mean. For ease of comparison, we calculated the natural logarithm of the response ratio (lnr). The standard deviation (SD) and the sample size (*n*) for each observation were collected to calculate the variance of the effect size.

The lnr was calculated without and with being standardized by warming magnitude (Equations 1, 2).

(1) $${\text{log}}_{e}r={\text{log}}_{e}\left(\frac{X_{t}}{X_{c}}\right)={\text{log}}_{e}\left(X_{t}\right)-{\text{log}}_{e}\left(X_{c}\right)$$

(2) $${\text{log}}_{e}r=\frac{{\text{log}}_{e}\left(\frac{X_{t}}{X_{c}}\right)}{T_{t}-T_{c}}=\frac{{\text{log}}_{e}\left(X_{t}\right)}{T_{t}-T_{c}}-\frac{{\text{log}}_{e}\left(X_{c}\right)}{T_{t}-T_{c}}$$

where T_t_ and T_c_ are the temperature in the warming and control treatments, respectively.

Using METAWIN software 2.1 (Sinauer Associates, Inc. Sunderland, MA, USA), we calculated the effect size of the target variables and used a weighted fixed-effect model to assess the effect of plant functional types, experimental settings, and treatment duration. If the 95% confidence interval (CI) of the effect size produced by the fixed-effect model overlaps with 0, no significant effect was detected on the response variables. If the upper limit of 95% CI is less than 0, the effect is considered significantly negative. In contrast, if the lower limit of 95% CI is greater than 0, the effect is considered significantly positive. If the 95% CI of the effect size among different species, pot size, and treatment duration does not overlap, their response is considered significantly different. Unless otherwise indicated, significance level was set at *p* < 0.05. The publication bias for effect size (lnr) in this meta-analysis was also calculated. We calculated Spearman's rank order correlation (rs) which indicates the relationship between the effect size (lnr) and the sample size (Begg and Mazumdar, 1994), and Rosenthal's fail-safe number which represents the number of additional studies with a mean effect size of zero needed to eliminate the significance of a significant effect (Rosenthal, 1979). Publication bias was significant if *p*-value of rs was smaller than 0.05. However, the publication bias may be safely ignored if the fail-safe number is larger than a critical value of 5n+10 where n is the number of studies (Rosenberg, 2005).

### Statistical Analysis

Original data collected from these studies were arranged into a database in which the value of response variables was lnr. The effect of warming duration on lnr was considered significant if the 95% confidence interval (CI) of lnr does not overlap with 0. And when the 95% confidence intervals (CI) of lnr of different PFTs, facilities or pot size did not overlap with each other, the response was considered significantly different among different categories, the means of the ratio of the R_d_/A_net_ and A_net_/G_s_ in the control and warming treatments were compared using paired *t*-test. The relationship between lnr of all the variables and the magnitude of warming treatments were evaluated by a second-degree polynomial or linear regression analysis with the R statistical programming language (R 3.2.2 for Windows GUI front-end).

## Results

### Effects of the Duration of Warming Treatment on Plant Ecophysiological Traits Across Plant Functional Types (PFTs) and Growth Forms

**Figure 1:**
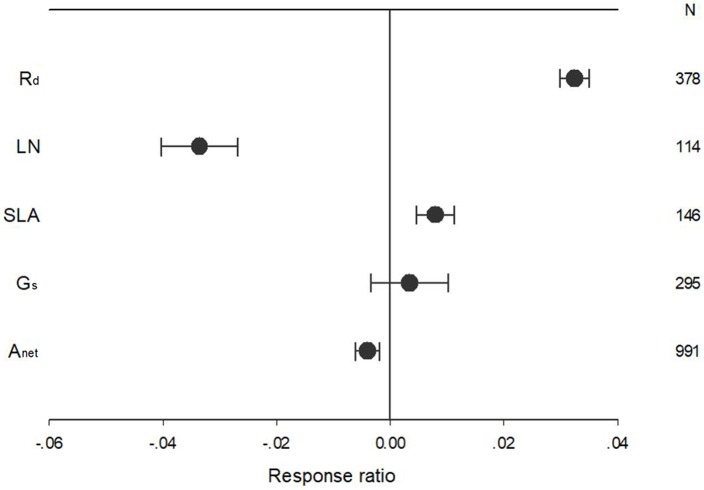
Ecophysiological responses of net photosynthetic rate (A_net_), stomatal conductance (G_s_), leaf nitrogen content (LN), specific leaf area (SLA), and leaf dark respiration rate (R_d_) to increased temperature. Each data point represents the mean ± 95% CI. The number of observations for each variable is given on the right of the graph.

**Figure 2:**
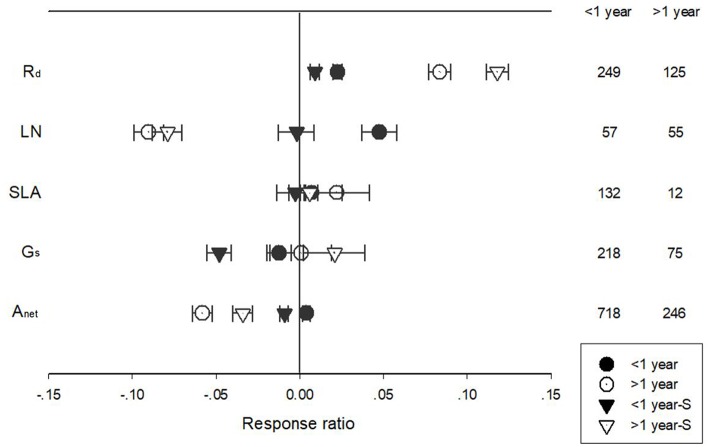
Standardized (triangle symbols) and unstandardized (circle symbols) responses of net photosynthetic rate (A_net_), stomatal conductance (G_s_), leaf nitrogen content (LN), specific leaf area (SLA), and leaf dark respiration rate (R_d_) to <1 year (closed symbols) and >1 year (open symbols) temperature treatment durations. Each data point represents the mean±95% CI. The number of observations for each variable is given on the right of the graph.

Warming treatment increased dark respiration (R_d_) and specific leaf area (SLA) and decreased net photosynthetic rate (A_net_) and leaf N concentration (LN) across all the experiments (Figure 1). The response of standardized (triangle symbols) or unstandardized (circle symbols) rate of A_net_, G_s_, R_d_, LN, and SLA to warming treatment differed with different warming durations (Figure 2). Long-term warming treatment (>1 year) had a greater positive effect on R_d_ than short-term (<1 year), regardless of whether the effect was standardized or unstandardized. LN was decreased by long-term warming but was increased or not changed by short-term warming treatment for unstandardized and standardized effect, respectively. Long-term warming treatment increased SLA, while short-term treatment had no effect on SLA. For standardized response of SLA, there was no difference between long and short-term treatments. For G_s_, long term treatment had a positive but short-term treatment had a negative effect on the standardized effect size. However, for the unstandardized effect size, short-term treatment did not have a significant but long-term had a negative effect on G_s_. Short-term had a positive and long-term treatment a negative effect on A_net_ for the unstandardized form of the effect. And for standardized effect of A_net_, long-term treatment had a more negative effect than short-term treatment (Figure 2).

**Figure 3:**
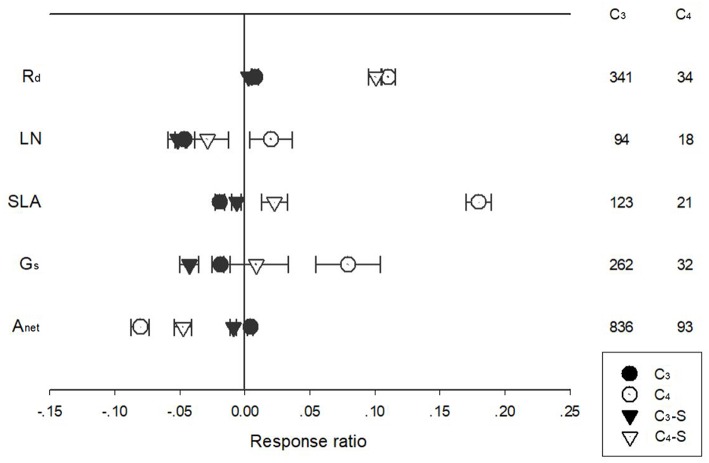
Standardized (triangle symbols) and unstandardized (circle symbols) of net photosynthetic rate (A_net_), stomatal conductance (G_s_), leaf nitrogen content (LN), specific leaf area (SLA), and leaf dark respiration rate (R_d_) of C_3_ (closed symbols) and C_4_ (open symbols) species to increased temperatures. Each data point represents the mean±95% CI. The number of observations for each variable is given on the right of the graph.

The response of A_net_, G_s_, R_d_, LN, and SLA to warming treatment differed among PFTs with different photosynthetic pathways (Figure 3). Warming had a more positive effect on R_d_ for C_4_ species than for C_3_ species, regardless of whether the effect size was standardized. Warming had a negative effect for C_3_ but a positive effect for C_4_ species on LN, SLA, and G_s_. In contrast, warming had a negative effect for C_4_ but near-zero effect for C_3_ species on A_net_ (Figure 3).

**Figure 4:**
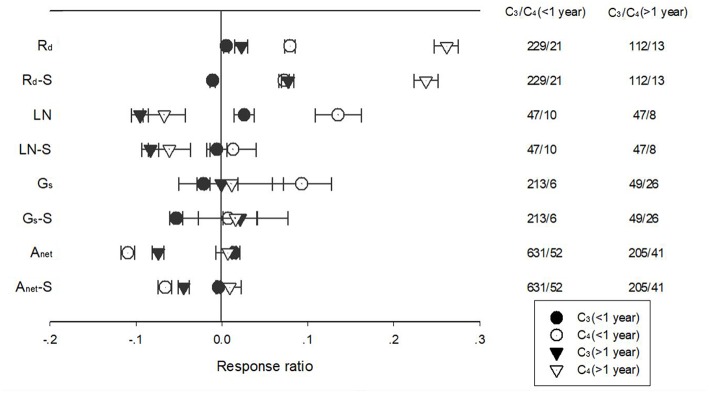
Responses of net photosynthetic rate (A_net_), stomatal conductance (G_s_), leaf nitrogen content (LN), specific leaf area (SLA) and leaf dark respiration rate (R_d_) of C_3_ (closed symbols) and C_4_ (open symbols) species to <1 year (circle symbols) and >1 year (triangle symbols) temperature treatment. Each data point represents the mean±95% CI. The number of observations for each variable is given on the right of the graph.

Warming duration had a significant effect on the response of A_net_, G_s_, R_d_, LN, and SLA for PFTs with different photosynthetic pathways (Figure 4). Long term warming treatment had a more positive effect than short-term on R_d_ for both C_3_ and C_4_ species, regardless of whether the effect was standardized. For LN, long term treatment had a negative effect but short-term treatment had a positive effect for C_3_ and C_4_ species. For C_3_ species, short term warming treatment had a positive and long-term had a negative effect on A_net_; for C_4_ species, long term warming treatment had a positive but short term a negative effect on A_net_. Similar trend was found for standardized A_net_, even though the magnitude of the effect differed.

### Effects of Warming Duration on Plant Traits Across Different Experimental Settings

**Figure 5:**
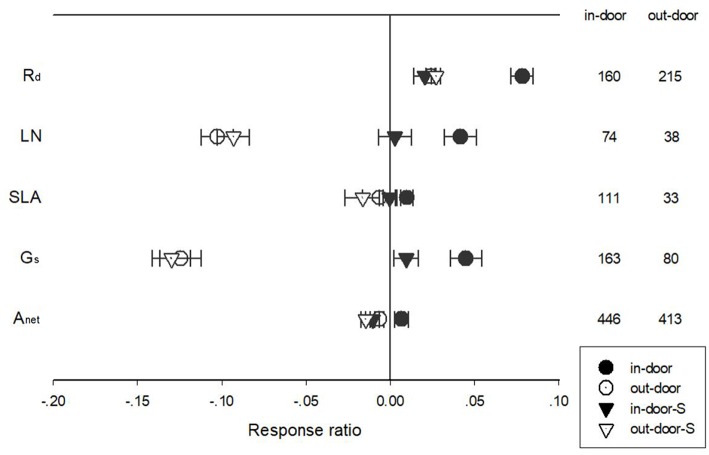
Standardized (triangle symbols) and unstandardized (circle symbols) responses of net photosynthetic rate (A_net_), stomatal conductance (G_s_), leaf nitrogen content (LN), specific leaf area (SLA), and leaf dark respiration rate (R_d_) to increased temperatures at in-door (closed symbols) and out-door (open symbols) experimental settings. Each data point represents the mean±95% CI. The number of observations for each variable is given on the right of the graph.

The responses of A_net_, G_s_, R_d_, LN, and SLA to warming treatment differed among in-door and outdoor experimental settings (Figure 5). Warming had a more positive impact on R_d_ in the in-door than the out-door settings for unstandardized effect size. Warming had a positive effect on LN for in-door, but a negative effect for outdoor settings. Being standardized with temperature treatment, warming had no impact on LN for the in-door but negative impact on outdoor experimental settings. Warming had a positive effect on SLA for in-door settings but tended to have a negative effect for outdoor settings. G_s_ responded positively to warming under in-door but negatively under outdoor settings. Warming treatment had a positive effect on unstandardized A_net_ under in-door settings but a negative effect under outdoor settings. For standardized A_net_, warming had a negative effect for both in-door and outdoor settings (Figure 5).

**Figure 6:**
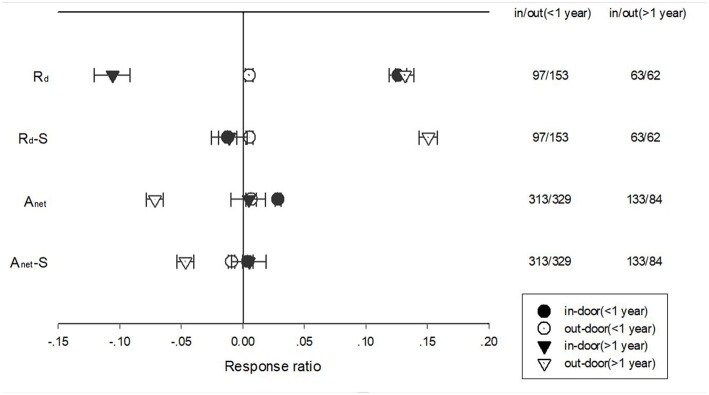
Responses of net photosynthetic rate (A_net_) and leaf dark respiration rate (R_d_) to <1 year (circle symbols) and >1 year (triangle symbols) temperature treatment at in-door (closed symbols) and out-door (open symbols) experimental settings. Each data point represents the mean±95% CI. The number of observations for each variable is given on the right of the graph.

The response of A_net_, G_s_, R_d_, LN, and SLA to warming treatment under indoor and outdoor experiment settings also differed with different treatment durations (Figure 6). Short-term warming had a positive effect but long-term had a negative effect on R_d_ for indoor experimental settings. Long-term warming had a more positive impact on R_d_ than short-term for outdoor experimental settings for both standardized and unstandardized effect size. Short-term warming treatment had a more positive impact than long-term treatment on A_net_ for unstandardized effect size but had no difference on standardized effect size. Short-term had a positive impact on A_net_ for outdoor settings, but long-term treatment had a negative impact on A_net_ for unstandardized effect. Long-term warming treatment had a more negative effect on standardized A_net_ than short term for outdoor settings (Figure 6).

**Figure 7:**
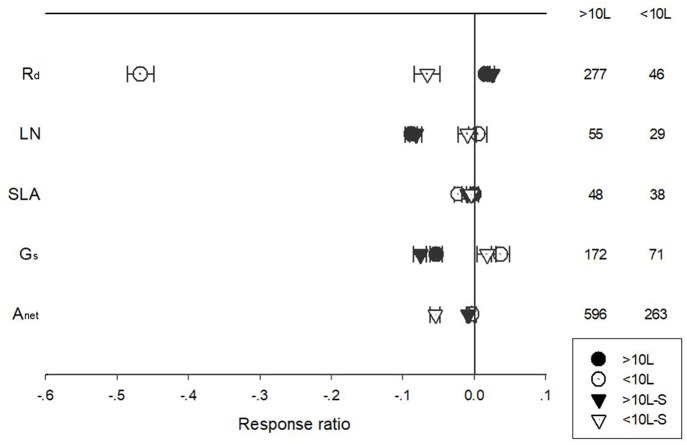
Standardized (triangle symbols) and unstandardized (circle symbols) responses of net photosynthetic rate (A_net_), stomatal conductance (G_s_), leaf nitrogen content (LN), specific leaf area (SLA), and leaf dark respiration rate (R_d_) to increased temperatures for plants grown at <10 L (closed symbols) and >10 L pots (open symbols). Each data point represents the mean±95% CI. The number of observations for each variable is given on the right of the graph.

Pot size had a significant impact on the responses of A_net_, G_s_, R_d_, and LN to warming treatment (Figure 7). Warming had a positive impact on R_d_ for plants grown in pots larger than 10 L, while a negative effect for plants grown in pots smaller than 10 L. G_s_ responded positively to warming when grown at <10 L plots but negatively at >10 L plots. A_net_ of plants grown at >10 L pots responded negatively to warming. Warming had no impacts on unstandardized A_net_ but a negative effect on standardized A_net_ of plants grown at <10 L pots.

**Figure 8:**
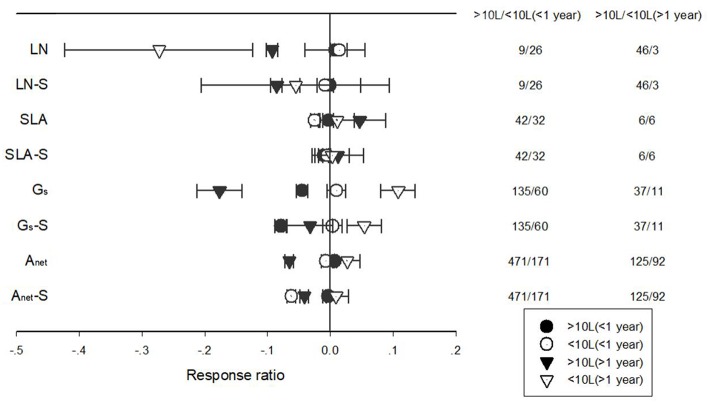
Responses of net photosynthetic rate (A_net_), stomatal conductance (G_s_), leaf nitrogen content (LN), specific leaf area (SLA), and leaf dark respiration rate (R_d_) to <1 year (circle symbols) and >1 year (triangle symbols) temperature treatment at in-door (closed symbols) and out-door (open symbols) experimental settings. Each data point represents the mean±95% CI. The number of observations for each variable is given on the right of the graph.

The response of A_net_, G_s_, LN, and SLA to warming treatment differed among different treatment durations when plants were grown in pots of different volumes (Figure 8). Short-term warming had a positive effect but long-term, a positive effect on LN for plants grown at both <10 L and >10 L pots. Short-term warming had a negative effect on SLA, but long-term a positive effect for both <10 L and >10 L pots. G_s_ responded positively with both short and long-term warming treatments at <10 L pots but negatively at >10 L pots. A_net_ responded positively to long-term warming treatment at <10 L pots but negatively at >10 L pots (Figure 8).

### Effects of Warming Magnitude on Plant Traits Across Different Experimental Settings

**Figure 9:**
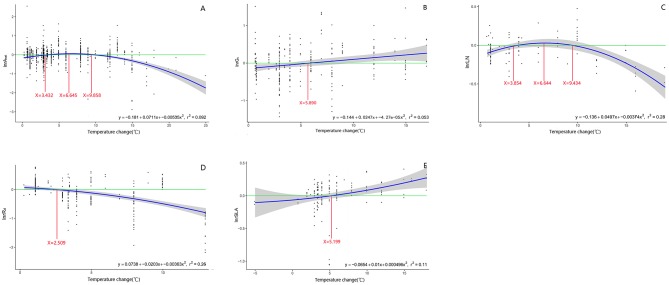
Regression relationship between the magnitude of warming treatment and the effect size of net photosynthetic rate (**A:** A_net_), stomatal conductance (**B:** G_s_), leaf nitrogen content (**C:** LN), specific leaf area (**D:** SLA), and leaf dark respiration rate (**E:** R_d_). Regression equation and variation coefficient are presented in the lower right corner of each graph. Different lines indicate x-value when y is the maximum (red line), crossing points of y = 0 (green line) and regression relationships (blue line).

A_net_, R_d_, LN, and SLA formed a quadratic relationship to warming treatment (Figure 9). The effect size of A_net_, R_d_, LN, and SLA to warming was highest or lowest when temperature change was 6.6, 2.5, 6.6, and 5.2°C above ambient temperature, respectively (Figure 9).

## Discussion

Several meta-analyses have investigated the general tendency of warming impacts on plant physiology and production (Rustad et al., 2001; Jing et al., 2016). However, it remains unclear how the experimental methodology of warming treatment affects the responses of plant ecophysiological traits to warming at leaf level. In this study, we collected data from warming manipulative studies and analyzed changes in the ecophysiological responses in the leaf traits. Overall, we found that (1) the direction and degree of the effect of warming treatment of different durations and settings on plant ecophysiological traits varied significantly; (2) there were significant variations among plant functional types in response to warming treatment of different methodology.

Consistent with previous findings from other studies, this meta-analysis confirmed that R_d_ and SLA were stimulated by warming treatment (Rustad et al., 2001; Jing et al., 2016). Increasing, decreasing or neutral impacts of experimental warming have been observed for net photosynthetic rates (Bruhn et al., 2007; Bronson and Gower, 2010; Li et al., 2013). The net photosynthetic rate in this analysis was significantly decreased by warming treatment. The decrease in plant photosynthetic capacity may be attributed to the decreased LN under warmed conditions. Many studies showed that plant photosynthetic capacity was positively related to leaf N concentrations (Kattge et al., 2009; Reich et al., 2009). Compared with the negative effect of warming for non-legumes, there was a positive or neutral effect on LN and A_net_ for legume species (Supplementary Material S3). Contrary to the expectations, stomatal conductance remained unchanged under warming, thus highlighting the key roles of biochemical and nutritional limitations on the negative responses of net photosynthesis to warming treatment. The response of G_s_ to global warming is critical for modeling ecosystem and landscape-scale water fluxes and CO_2_ exchange. The ratio of respiration to photosynthesis (*R/P*) has been used to express the proportion of consumed to fixed C of plants (Atkin et al., 2007; Campbell et al., 2007) and shown to be enhanced (Danby and Hik, 2007; Wan et al., 2009), suppressed (Jochum et al., 2007), or maintained (He et al., 2005) by experimental warming. The ratio of R_d_/A_net_ was increased at warming conditions (effect size is 0.3623, *n* = 275) in this study, suggesting that the respiration was more affected and a greater proportion of fixed C was consumed, implying a decline of the net amount of C fixed by leaves by warming, at least in the controlled experiments.

Ecophysiological traits responses of terrestrial plants to increased temperature varied among plant functional types with different photosynthetic pathways (PFTs; Wang et al., 2012; Jing et al., 2016). Previous studies indicated that global warming had stronger effects on A_net_ of C_3_ species than C_4_ species (Wahid et al., 2007). In this study, the positive and negative effects of warming on R_d_ and A_net_ were greater for C_4_ species than C_3_ species, in spite of positive or neutral effects of warming on LN, SLA, and G_S_ for C_4_ and C_3_ species, respectively. The contradictory findings posed great challenges for projecting the responses and feedbacks of terrestrial ecosystems to global warming. The more disadvantaged situation for C_4_ species under warming might be associated with higher growth and treatment temperature applied in the experiment (Supplementary Material S1). The metabolic balance of the photosynthetic and respiratory processes under climate warming plays a critical role in regulating ecosystem carbon storage and cycling (Schimel, 1995; King et al., 2006).

Warming stimulated A_net_ in woody but suppressed it in herbaceous plants (Supplementary Material S4). The positive effect of warming on A_net_ for woody species was unrelated to either G_s_ or LN, as G_s_ and LN both were decreased under warming treatments (Supplementary Material S4). The results from this study were similar to the trend reported for trees showing a lower percentage decrease in G_s_ compared to herbaceous species (Wang et al., 2012). Warming had a positive effect on G_s_ and LN for crops, while a negative effect for non-crops (Supplementary Material S5). The changes in G_s_ at warming treatment may alter leaf temperature and result in a change in latent heat loss through evaporation, which may further affect net carbon balance (Warren et al., 2011). Warming could influence vegetation dynamics and ecosystem structure through shifting competitive interactions among different functional groups in natural or agricultural systems. Therefore, knowledge of photosynthetic and stomatal responses to increased temperature of different PFTs instead of species will facilitate the prediction of terrestrial C- and water- cycle feedback to climate warming.

Ecophysiological trait responses of terrestrial plants to increased temperature varied among warming treatments of differing durations. The physiological acclimation can lead to smaller enhancements of plant photosynthesis and respiration under long term warmer conditions than predicted with photosynthesis/respiration-temperature relationships (Medlyn et al., 2002; Dwyer et al., 2007; Tjoelker and Zhou, 2007; Gunderson et al., 2010). The thermal acclimation of R_d_ could minimize the effects of climate warming on C loss via plant respiration (Gifford, 1995; Ziska and Bunce, 1998; Loveys et al., 2002) and mitigate the positive feedback between climate change and atmospheric CO_2_ (King et al., 2006; Atkin et al., 2008). The findings in this meta-analysis indicated that the negative effect of warming treatment on A_net_ and LN and the positive effect on R_d_ were more evident under >1 year warming treatment and the trend was confirmed for both C_3_ and C_4_ species (Figure 4), which contrasted to other studies showing significant declines in the photosynthetic and/or respiratory response with increasing exposure time, a thermal acclimation to warming (Hikosaka et al., 2006; Gunderson et al., 2010).

Potential confounding factors must be accounted in the meta-analysis because many studies were conducted under variable conditions and targeted on different species. In this analysis, studies in which plants were grown under other environmental stresses such as drought, low nutrients, light deficiency or elevated ozone were excluded. In addition to the variation caused by plant functional types and treatment duration, different experimental facilities could be responsible for the responses of different PFTs (Cheesman and Klaus, 2013; Rehmani et al., 2014). This study mainly focused on the effects of pot size (<10 L vs. >10 L) and experimental settings (in-door vs. out-door) on plant ecophysiological responses. Warming had a negative and positive effect on LN and G_s_ when plants were grown at outdoor and in-door settings, respectively. Pot size significantly altered the responses of R_d_, LN and SLA to warming treatments. Warming had a negative effect on R_d_ for plants grown at <10 L pots, while a positive effect at >10 L pot. For both LN and G_s_, warming had a negative effect for plants grown at >10 L pots, while a neutral effect at <10 L pot. We were expecting that warming would have a more negative effect on LN and G_s_ in smaller pots or in-door settings considering that below-ground growth would be more constrained and thus limited the nutrients and water supply to the aboveground growth (Walters and Reich, 1989; Climent et al., 2011), the analysis indicated that this was true only when experiment duration was longer than 1 year when negative effects of warming was more evident for plants grown at <10 L pots.

Warming treatment duration had a significant interactive effect with experimental settings (in-door vs. outdoor) on R_d_ and A_net_. Long-term warming had a negative effect on R_d_ for in-door and on A_net_ for outdoor experimental settings. The findings in this meta-analysis indicated that the negative effect of warming treatment on A_net_ and LN and the positive effect on R_d_ were more evident under >1 year warming treatment and the trend was confirmed for both C_3_ and C_4_ species (Figure 4). The negative effect of warming on R_d_ could be related to the higher treatment temperature applied at the in-door settings (Supplementary Material S2). Temperature conditions in which plants live may be another possible reason for the contradictory findings (Rustad et al., 2001). The discrepancy of the response of A_net_ and R_d_ to warming treatment under different experimental settings provided difficulty in parameterizing ecosystem models and raised concerns in proper experimental designs when dealing with climate change questions.

The intensities of temperature treatment also had a significant impact on most of the parameters that were investigated in the study. The effect size of A_net_, R_d_, LN, and SLA responded to temperature increase in a quadratic relationship. Consistent with the results discussed before, the peak value of the ecophysiological traits of A_net_, R_d_, and LN occurred at temperatures higher than the ambient. Plant physiological responses to warming may also depend on the temperature regime they are grown at. Studies often report a positive response to warming in Rubisco carboxylation, photosynthesis, and growth in cool-climate species but reduced growth and carbon gain in species that exist in warm low-latitude climates (Way and Oren, 2010; Crous et al., 2018).

## Conclusion

Overall, we found that warming treatment of different durations and settings had different impacts on plant ecophysiological traits and the responses varied significantly among different plant functional types. Warming stimulated R_d_ and SLA but suppressed A_net_ and LN and the effect varied among different PFTs and experimental designs. The positive and negative effects of warming on R_d_ and A_net_, were greater for C_4_ than C_3_ species, in spite of the positive or neutral effects of warming on LN, SLA, and G_S_ for C_4_ and C_3_ species, respectively. The findings in this meta-analysis also indicated that the negative effect of warming treatment on A_net_ and LN and the positive effect on R_d_ were more evident under >1 year warming treatment and the trend was confirmed for both C_3_ and C_4_ species. Negative effect of warming was more evident for plants grown at <10 L pots only when experiment duration was longer than 1 year. The magnitude of temperature treatment did have an impact on most of the parameters that were investigated in the study. The functional type specific response patterns of plant traits to warming are critical for obtaining credible predictions of the changes in food production, carbon sequestration and climate regulation. This result also highlights the need for cautiously selecting parameter values in forecasting ecosystem function changes in future climate regimes, evaluating much more broadly what can and cannot be learned from experimental studies and designing controlled experiments to realistically reflecting ecosystems responses to future global warming.

## Data Availability

All datasets for this study are included in the manuscript and the supplementary files.

## Author Contributions

DW and ZY conceived and wrote the paper. The rest of the authors helped collection data and ran data analysis.

### Conflict of Interest Statement

The authors declare that the research was conducted in the absence of any commercial or financial relationships that could be construed as a potential conflict of interest.
