# Supplementary material for: Warming Treatment Methodology Affected the Response of Plant Ecophysiological Traits to Temperature Increases: A Quantitive Meta-Analysis
Source: Front Plant Sci. 2019 Sep 6;10:957. doi: 10.3389/fpls.2019.00957 (PMC6743343; doi:10.3389/fpls.2019.00957)

**Supplementary materials**

**S1:** The data information and journal articles from which data were collected.

| *Species* | Temperature change | in-door or outdoor | Pot size | Photosynthetic pathway | Measuring  instrument | Measuring  Light level | Measuring date | Legumes or non-legumes | Wood or herbaceous | Crop or non-crop | Citation |
| --- | --- | --- | --- | --- | --- | --- | --- | --- | --- | --- | --- |
| *A. faxoniana* | 2.2℃ | in | <10L | C3 | LI-6400 | NA | NA | non-leg | wood | wild | Wang J.C.et al.2012 |
| *A. holophylla* | 3℃ | out | >10L | C3 | CI-340 | 1000 | June to October 2012 | non-leg | wood | wild | Han S.et al.2015 |
| *A. koreana* | 3℃ | out | >10L | C3 | CI-340 | 1000 | June to October 2012 | non-leg | wood | wild | Han S.et al.2015 |
| *A.spicatum L.* | 5℃ | in | <10L | C3 | NA | NA | NA | non-leg | wood | wild | Danyagri G.et al.2014 |
| *Agropyron cristatum* | 11.7℃ | out | >10L | C3 | LI-6400 | 1500 | 2005 to 2012 | non-leg | herb | wild | Song B.et al.2016 |
| *Agropyron cristatum* | 2.9℃ | in | <10L | C3 | LI-6400 |  | Between April 2011 and July 2013 | non-leg | herb | wild | Valencia E.et al.2016 |
| *Agropyron cristatum* | 8℃ | out | >10L | C3 | LI-6400 | 1500 | July in 2005 | non-leg | herb | wild | Niu S.L.et al.2008 |
| *Alliaria petiolata* | 3℃ | in | <10L | C3 | LI-6400 | NA | June and September 2007 | non-leg | herb | wild | Anderson L.J.2013 |
| *Ambrosia psilostachya* | 1.1℃ | out | >10L | C3 | LI-6400 | 1000 | May to Sep | non-leg | herb | wild | Zhou X.H.et al.2007 |
| *Ambrosia pssilostachya* | 1.1℃ | out | >10L | C3 | NA | NA | NA | non-leg | wood | wild | An Y.et al.2005 |
| *Artemisia capillaris* | 8℃ | out | >10L | C3 | LI-6400 | 1500 | July in 2005 | non-leg | herb | wild | Niu S.L.et al.2008 |
| *Aspen(P.tremula)* | 0.8℃ | out | >10L | C3 | LI-6400 | 1200 | Jun Oct in 2007 | non-leg | wood | wild | Maenpaa M.et al.2011 |
| *Aster ericodies* | 1.1℃ | out | >10L | C3 | NA | NA | NA | non-leg | wood | wild | An Y.et al.2005 |
| *Aster ericoides* | 1.1℃ | out | >10L | C3 | LI-6400 | 1000 | 2000-2001 | non-leg | herb | wild | Zhou X.H.et al.2007 |
| *B.albo-sinensis* | 6℃ | in | <10L | C3 | NA | NA | NA | non-leg | wood | wild | Duan B.L.et al.2013 |
| *Betula nana* | 0.9℃ | in | <10L | C3 | NA | NA | NA | non-leg | wood | wild | Dorrepaal E.et al.2006 |
| *Betula papyrifera* | 3℃ | in | <10L | C3 | NA | NA | NA | non-leg | wood | wild | Tjoelker M.G.et al.2009 |
| *Betula alleghaniensis* | 2℃ | in | >10L | C3 | LI-6400 | 1500 | May and June in 2003 | non-leg | wood | wild | Gunderson C.A.et al.2010 |
| *Calamagrostis lapponica* | 0.9℃ | in | <10L | C3 | NA | NA | NA | non-leg | wood | wild | Dorrepaal E.et al.2006 |
| *Caragana microphylla* | 6℃ | in | <10L | C3 | LI-6400 | 1000 | 2000-2001 | leg | wood | wild | Zhou X.H.et al.2007 |
| *Colobanthus quitensis* | 13℃ | in | <10L | C3 | LI-6400 | 750 | 85 days | non-leg | herb | wild | Xiong F.S.et al.2000 |
| *Deschampsia antarctica* | 5℃ | in | <10L | C3 | LI-6400 | 750 | 85 days | non-leg | herb | wild | Xiong F.S.et al.2000 |
| *Dichanthelium oligosnathes* | 1.1℃ | out | >10L | C3 | NA | NA | NA | non-leg | herb | wild | An Y.et al.2005 |
| *Dorycnium pentaphyllum* | 2.9℃ | in | <10L | C3 | LI-6400 |  | Between April 2011 and July 2013 | non-leg | herb | wild | Valencia E.et al.2016 |
| *E.paucifbra Sieb.ex Spreng.* | 2.2℃ | in | <10L | C3 | NA | NA | NA | non-leg | wood | wild | Duan B.L.et al.2013 |
| *Empetrum nigrum* | 0.9℃ | in | <10L | C3 | NA | NA | NA | non-leg | wood | wild | Dorrepaal E.et al.2006 |
| *Eucalyptus globulus Labill* | 3℃ | in | >10L | C3 | LI-6400 | 1800 | NA | non-leg | wood | wild | Quentin A.G.et al.2015 |
| *Eucalyptus grandis* | 3.5℃ | out | >10L | C3 | LI-6400 | 1800 | NA | non-leg | wood | wild | Drake J.E.et al.2015 |
| *Eucalyptus tereticornis* | 3℃ | out | >10L | C3 | LI-6400 | 1800 | NA | non-leg | wood | wild | Drake J.E.et al.2015 |
| *Eurybia macrophylla* | 3.4℃ | out | >10L | C3 | LI-6400 | 800 | NA | non-leg | herb | wild | Jacques M.H.et al.2015 |
| *Festuca ovina* | 2.9℃ | in | <10L | C3 | LI-6400 | NA | Between April 2011 and July 2013 | non-leg | herb | wild | Valencia E.et al.2016 |
| *Gentiana straminea* | 15℃ | out | >10L | C3 | LI-6400 | 1500 | NA | non-leg | herb | wild | Shen H.H.et al.2013 |
| *Geum vernum* | 3℃ | in | <10L | C3 | LI-6400 | NA | NA | non-leg | herb | wild | Anderson L.J.2013 |
| *H.squamatum* | 2℃ | in | <10L | C3 | LI-6400 | 1500 | NA | non-leg | herb | wild | Leon S.L.et al.2016 |
| *Hedysarum coronarium* | 2.9℃ | in | <10L | C3 | LI-6400 | NA | Between April 2011 and July 2013 | non-leg | herb | wild | Valencia E.et al.2016 |
| *Helianthus mollis Lam* | 1.1℃ | out | >10L | C4 | LI-6400 | 1000 | NA | non-leg | herb | wild | Zhou X.H.et al.2007 |
| *Icatu* | 17℃ | in | >10L | C3 | LI-6400 | 1200 | NA | non-leg | wood | wild | Rodrigues W.P.et al.2016 |
| *IPR108* | 17℃ | in | >10L | C3 | LI-6400 | 1200 | NA | non-leg | wood | wild | Rodrigues W.P.et al.2016 |
| *Lantana camara* | 4℃ | in | <10L | C3 | LI-6400 | 1200 | NA | non-leg | wood | wild | Zhang Q.Y.et al.2014 |
| *Larix laricina* | 12℃ | in | <10L | C3 | NA | NA | NA | non-leg | wood | wild | Tjoelker M.G.et al.2009 |
| *Leymus chinensis* | 6℃ | in | <10L | C3 | LI-6400 | NA | NA | non-leg | herb | wild | Song X.L.et l.2016 |
| *Liquidambar styracflua* | 4℃ | in | >10L | C3 | LI-6400 | 1500 | NA | non-leg | wood | wild | Gunderson C.A.et al.2010 |
| *Lygeum spartum* | 2.9℃ | in | <10L | C3 | LI-6400 | NA | Between April 2011 and July 2013 | non-leg | herb | wild | Valencia E.et al.2016 |
| *Maianthemum canadense* | 3.4℃ | out | >10L | C3 | LI-6400 | 800 | NA | non-leg | herb | wild | Jacques M.H.et al.2015 |
| *maize* | 2.7℃ | out | >10L | C3 | LI-6400 | 1500 | NA | non-leg | herb | crop | Ruiz-Vera U.M.et al.2015 |
| *Medicago sativa* | 2.9℃ | in | <10L | C3 | LI-6400 | NA | Between April 2011 and July 2013 | non-leg | herb | wild | Valencia E.et al.2016 |
| *P.densiflora* | 3℃ | out | >10L | C3 | CI-340 | 1000 | NA | non-leg | wood | wild | Han S.et al.2015 |
| *P.koraiensis* | 3℃ | out | >10L | C3 | CI-340 | 1000 | NA | non-leg | wood | wild | Han S.et al.2015 |
| *P.cathayana* | 1.99℃ | in | >10L | C3 | LI-6400 | 1400 | NA | non-leg | wood | wild | Xu X.et al.2010 |
| *P.densiflora* | 3℃ | out | >10L | C3 | CIRAS-2 | 1100 | NA | non-leg | wood | wild | Yun S.J.et al.2016 |
| *Pennisetum centrasiaticum* | 8℃ | out | >10L | C3 | LI-6400 | 1500 | NA | non-leg | herb | wild | Niu S.L.et al.2008 |
| *Phlomis herba-venti* | 2.9℃ | in | <10L | C3 | LI-6400 |  | Between April 2011 and July 2013 | non-leg | herb | wild | Valencia E.et al.2016 |
| *Phragmites australis* | 4℃ | in | >10L | C3 | NA | NA | NA | non-leg | herb | wild | Flury S.et al.2014 |
| *Picea abies* | 8℃ | in | >10L | C3 | LI-6400 | 1200 |  | non-leg | wood | wild | Kroner Y.et al.2016 |
| *Picea mariana* | 12℃ | in | <10L | C3 | NA | NA | NA | non-leg | wood | wild | Tjoelker M.G.et al.2009 |
| *Pinus banksiana* | 12℃ | in | <10L | C3 | NA | NA | NA | non-leg | wood | wild | Tjoelker M.G.et al.2009 |
| *Plantago lanceolata* | 2.9℃ | in | <10L | C3 | LI-6400 | NA | Between April 2011 and July 2013 | non-leg | herb | wild | Valencia E.et al.2016 |
| *Populus tremuloides* | 6℃ | in | <10L | C3 | NA | NA | NA | non-leg | wood | wild | Tjoelker M.G.et al.2009 |
| *Populus grandidentata* | 4℃ | in | >10L | C3 | LI-6400 | 1500 | NA | non-leg | wood | wild | Gunderson C.A.et al.2010 |
| *Potentilla acaulis* | 8℃ | out | >10L | C3 | LI-6400 | 1500 | NA | non-leg | herb | wild | Niu S.L.et al.2008 |
| *Psoralea bituminosa* | 2.9℃ | in | <10L | C3 | LI-6400 | NA | Between April 2011 and July 2013 | non-leg | herb | wild | Valencia E.et al.2016 |
| *Quercus rubra* | 4℃ | in | >10L | C3 | LI-6400 | 1500 | NA | non-leg | wood | wild | Gunderson C.A.et al.2010 |
| *Rubus chamaemorus* | 0.9℃ | in | <10L | C3 | NA | NA | NA | non-leg | wood | wild | Dorrepaal E.et al.2006 |
| *S.dulcamara* | 6℃ | in | <10L | C3 | LI-6400 | 1500 | NA | non-leg | wood | wild | Flynn D.F.B.et al.2006 |
| *Sanguisorba minor* | 2.9℃ | in | <10L | C3 | LI-6400 | NA | Between April 2011 and July 2013 | non-leg | herb | wild | Valencia E.et al.2016 |
| *Schizachyrium scopariums* | 1.1℃ | out | >10L | C4 | NA | NA | NA | non-leg | herb | wild | An Y.et al.2005 |
| *silver birch(Betula pendula)* | 0.8℃ | out | >10L | C3 | LI-6400 | 1200 | NA | non-leg | wood | wild | Maenpaa M.et al.2011 |
| *Sorghastrum nutans* | 1.1℃ | out | >10L | C3 | LI-6400 | 1000 | NA | non-leg | herb | wild | Zhou X.H.et al.2007 |
| *soybean* | 4℃ | in | >10L | C3 | LI-6400 | 1200 | NA | leg | herb | crop | Locke A.M.et al.2013 |
| *Spinach* | 14℃ | in | <10L | C3 | NA | NA | NA | non-leg | herb | crop | Holaday A.S.et al.1992 |
| *Sporobolus asper* | 1.1℃ | out | >10L | C3 | NA | NA | NA | non-leg | herb | wild | An Y.et al.2005 |
| *Stipa grandis* | 6℃ | in | <10L | C3 | LI-6200 | NA | NA | non-leg | herb | crop | Xu Z.Z.et al.2009 |
| *Stipa grandis* | 6℃ | in | <10L | C3 | LI-6400 | NA | NA | non-leg | herb | wild | Song X.L.et l.2016 |
| *Stipa krylovii* | 8℃ | out | >10L | C3 | LI-6400 | 1500 | NA | non-leg | herb | wild | Niu S.L.et al.2008 |
| *Stipa sareptana var.krylovii* | 11.7℃ | out | >10L | C3 | LI-6400 | 1500 | NA | non-leg | herb | wild | Song B.et al.2016 |
| *sugar maple* | 4℃ | out | >10L | C3 | LI-6400 | 1500 | NA | non-leg | wood | wild | Gunderson C.A.et al.2010 |
| *Triticum aestivum L.cv Yangmai 11* | 1.5℃ | out | >10L | C3 | LI-6400 | NA | NA | leg | herb | crop | Tian Y.L.et al.2012 |

**S2.** The frequency diagram of the warming treatment intensities used in the studies where the data were collected.


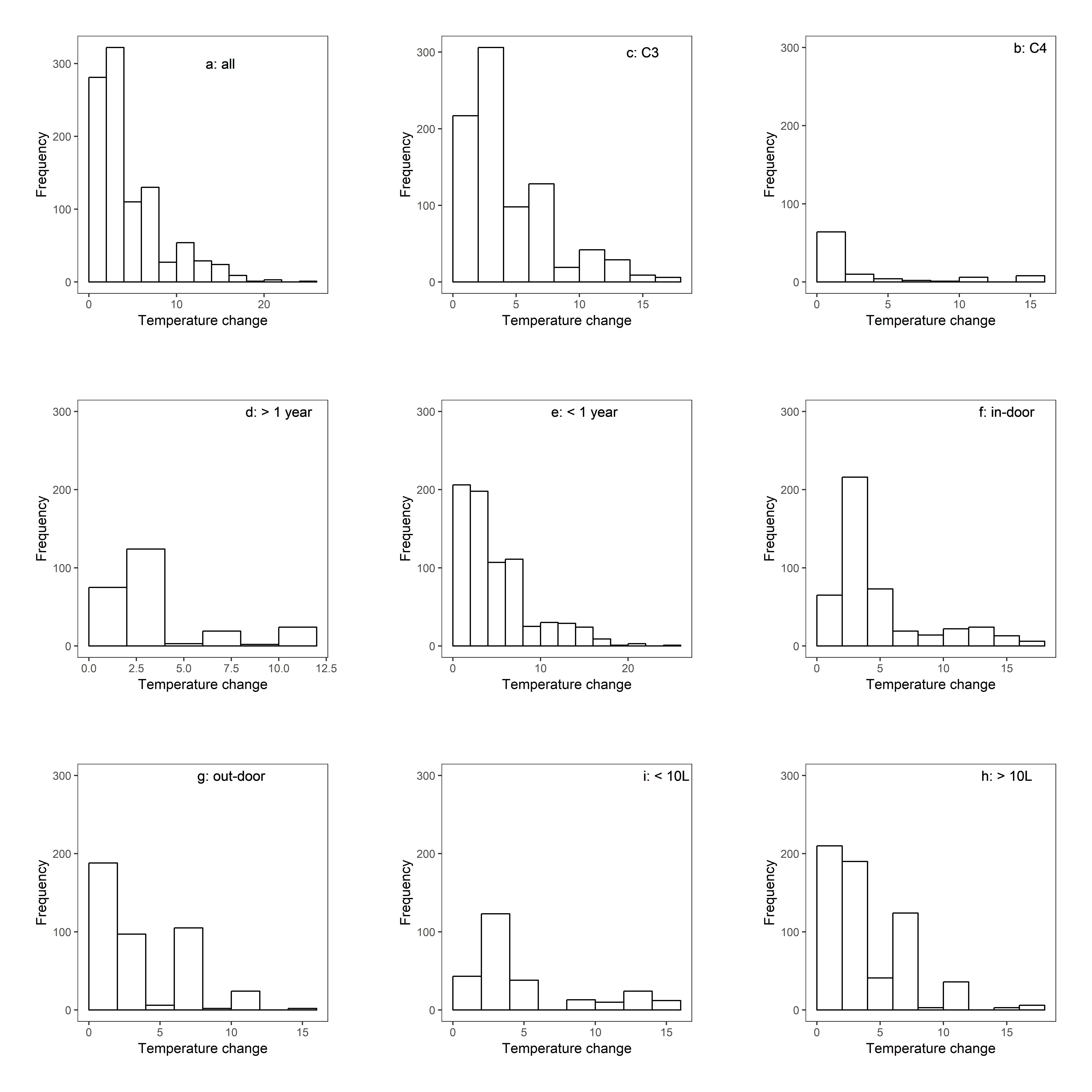


**S3.** Standardized (triangle symbols) and unstandardized (circle symbols) of net photosynthetic rate (A_net_), stomatal conductance (G_s_), leaf nitrogen content (LN), specific leaf area (SLA) and leaf dark respiration rate (R_d_) of legumes (closed symbols) and non-legume (open symbols) species to increased temperatures. Each data point represents the mean±95% CI. The number of observations for each variable is given on the right of the graph.


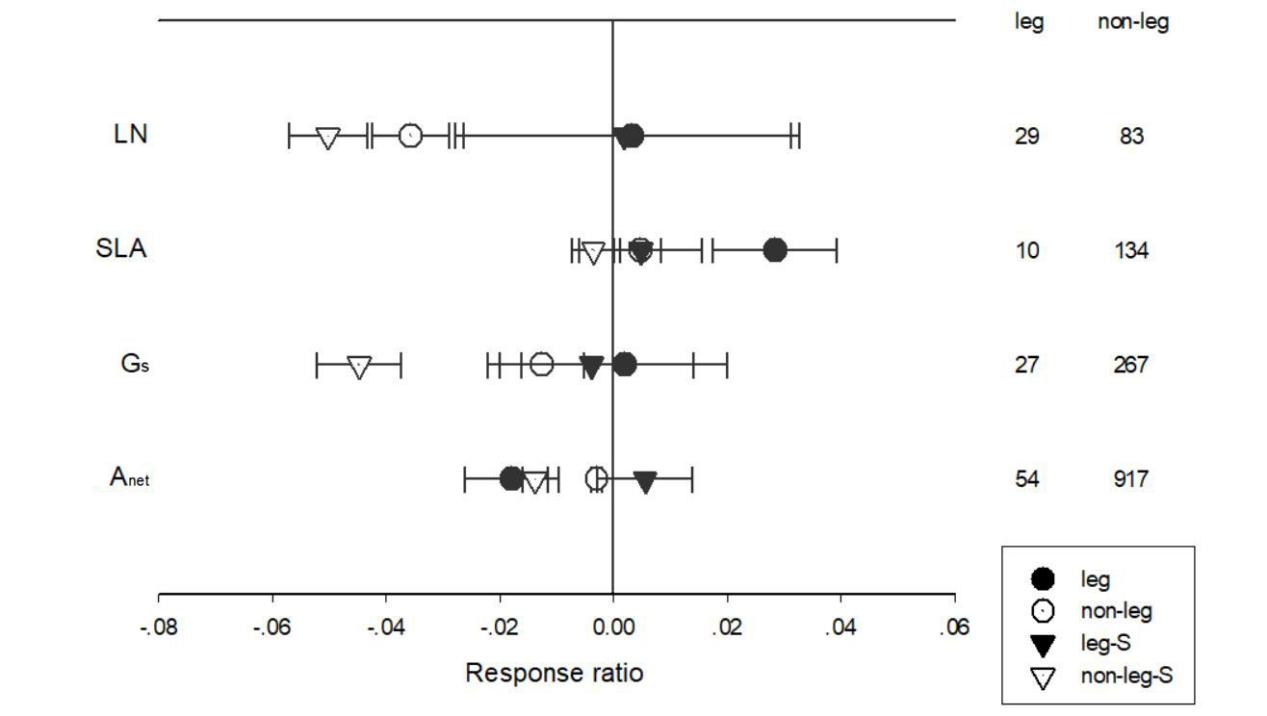


**S4.** Standardized (triangle symbols) and unstandardized (circle symbols) of net photosynthetic rate (A_net_), stomatal conductance (G_s_), leaf nitrogen content (LN), specific leaf area (SLA) and leaf dark respiration rate (R_d_) of woody (closed symbols) and herbaceous (open symbols) species to increased temperatures. Each data point represents the mean±95% CI. The number of observations for each variable is given on the right of the graph.Woody and herbaceous.


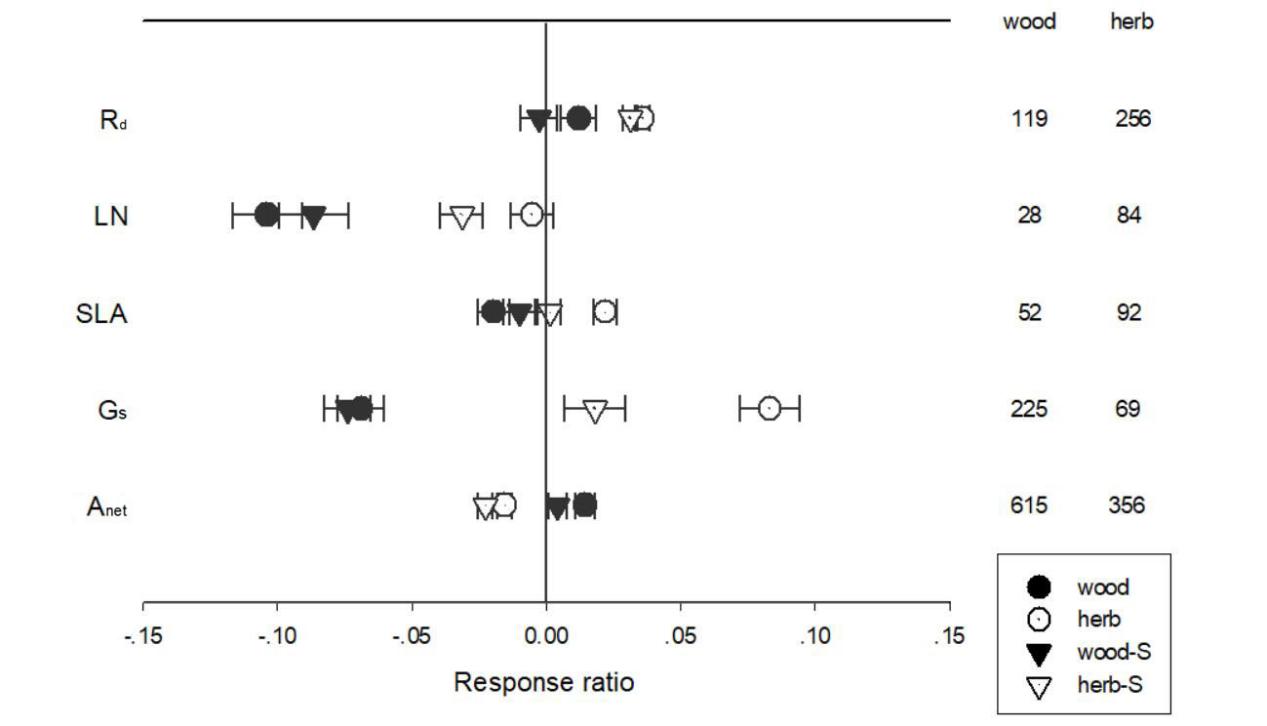


**S5.** Standardized (triangle symbols) and unstandardized (circle symbols) of net photosynthetic rate (A_net_), stomatal conductance (G_s_), leaf nitrogen content (LN), specific leaf area (SLA) and leaf dark respiration rate (R_d_) of wild (closed symbols) and crop (open symbols) species to increased temperatures. Each data point represents the mean±95% CI. The number of observations for each variable is given on the right of the graph.Woody and herbaceous.


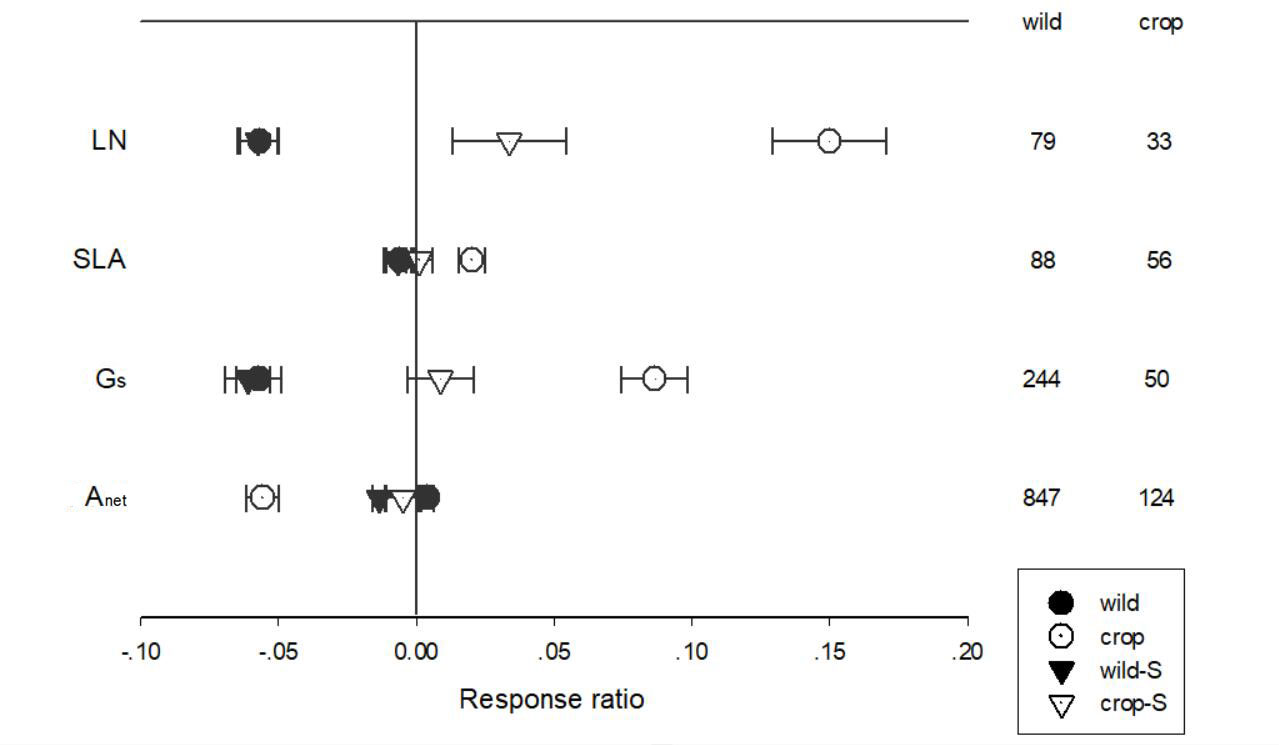

Supplement: Supplementary file 1 [file Data_Sheet_1.docx]
